# Postpartum Depressive Symptoms and Mother–Infant Bonding: A Mixed‐Methods Study Among Postpartum Women in Ghana

**DOI:** 10.1155/nrp/2803636

**Published:** 2026-07-31

**Authors:** Leticia Tornyevah, Jens Henrichs, Anjali Sharma, Ank De Jonge, Samuel Bosomprah

**Affiliations:** ^1^ Department of Midwifery Science, Amsterdam University Medical Centers, Vrije Universiteit Amsterdam, Amsterdam, the Netherlands, vu.nl; ^2^ Midwifery Academy Amsterdam Groningen, Inholland University of Applied Sciences, Amsterdam, the Netherlands, inholland.nl; ^3^ Amsterdam Public Health Research Institute, Amsterdam, the Netherlands, vumc.nl; ^4^ Department of Primary and Long-Term Care, University Medical Center Groningen, University of Groningen, Groningen, the Netherlands, rug.nl; ^5^ Research Department, Centre for Infectious Disease Research in Zambia, Lusaka, Zambia, cidrz.org; ^6^ Amsterdam Reproduction & Development Research Institute, Amsterdam, the Netherlands; ^7^ Department of Biostatistics, School of Public Health, University of Ghana, Accra, Ghana, ug.edu.gh

**Keywords:** Ghana, mother–infant bonding, postpartum depressive symptoms

## Abstract

Mother–infant bonding difficulties can impair the emotional connection between mother and child and affect child development. We examined the association between postpartum depressive symptoms (PPDS) and mother–infant bonding in the first year postpartum and explored sociocultural factors that may influence bonding. We employed a cross‐sectional mixed‐methods approach at three health facilities. Surveys included 399 mothers within 12 months postpartum. PPDS were assessed using the Patient Health Questionnaire‐9 with a cut‐off score > 9, and bonding was measured with the Pre‐ and Postnatal Bonding Scale. We used a log‐normal multivariable regression model to estimate the association between PPDS and mother–infant bonding, adjusting for potential confounders. Qualitative interviews were conducted with 31 mothers (19 with depressive symptoms and 12 without) and were analysed thematically. Overall, 29% of mothers screened positive for depressive symptoms. Screening positive was associated with a small, statistically significant reduction of 3% in bonding (adjusted mean ratio 0.97; 95% confidence interval 0.95–0.99; *p* = 0.01), among affected mothers compared to those not affected. However, the magnitude of this association was modest and should be interpreted with caution. Qualitative findings suggested that bonding appeared to be sustained through maternal instinct; caregiving; social support and religious, cultural and societal expectations in both groups, which may help explain the modest association observed. Culturally sensitive support and strengthened social networks may help mothers maintain strong bonds during the postpartum period.

## 1. Introduction

The development of mother–infant bonding is crucial during the postpartum period. Mother–infant bonding is characterised by the self‐perceived emotional connection between a mother and her child that stems from the caregiving system and helps protect the infant [1, 2]. It involves both affective and behavioural expressions such as holding, feeding and responsive interaction that nurture closeness and reinforce maternal identity [3]. Postnatal maternal bonding is associated with better infant attachment quality and mood, less infant temperament difficulties and lower colic rating [4]. However, several factors, for example, prematurity and lack of social support, can disrupt mother–infant bonding, leading to maternal bonding difficulties [5, 6]. Particularly, postpartum depression negatively affects the mother’s capability to develop a stable emotional bond with her infant among 6%–41% of mother–infant pairs [7, 8]. In Ghana, direct estimates of poor bonding are scarce; however, the high prevalence of maternal mental health conditions such as postpartum depression (affecting roughly one‐quarter of new mothers) suggests a substantial at‐risk population [9].

The early disruption in the mother–child bond carries serious consequences for child development, including elevated risks of poor infant behavioural and cognitive growth [4, 10]. Infants of mothers with bonding impairments show higher rates of failure to thrive, attachment and behavioural disorders and later learning problems [11]. Maternal well‐being is also compromised, as bonding impairments often co‐occur with postpartum depression or post‐traumatic stress symptoms, compounding the mother’s emotional distress [11, 12]. Mothers with postpartum depressive symptoms (PPDS) often report feeling irritable, anxious or emotionally withdrawn, reducing their sensitivity and responsiveness to the infant [13]. Previous research indicates that maternal depression may be a risk factor for bonding difficulties, as evidenced in one study where mothers scoring in the clinical range for PPDS were about five times more likely to report impaired bonding than nondepressed mothers [14, 15]. Other work has shown that depressed mothers tend to have consistently lower bonding scores over time than nondepressed mothers [14, 16]. At the same time, some evidence suggests that bonding can improve in all mothers as the infant grows. For example, one longitudinal study found that both depressed and nondepressed mothers showed increased bonding with their baby over the first 6 months postpartum, although the depressed group remained slightly behind [16]. In line with this, another study observed that PPDS’s negative effect on bonding was only apparent in the early postpartum months and disappeared by 1 year [14]. Furthermore, a systematic review reported no association between PPDS and mother–infant bonding [17]. This inconsistency highlights the need for further research, particularly in different cultural contexts such as Ghana, where social support and cultural norms may influence bonding [9].

Our study aimed to deepen the understanding of how PPDS affects mother–infant bonding in a sub‐Saharan African context. The insights gained are expected to inform targeted interventions and policies to support mother–infant bonding among women suffering from PPDS. The specific objectives were twofold. First, to determine the association between PPDS and the level of mother–infant bonding within the first year postpartum, and second, to qualitatively explore the lived experiences of postpartum depressed mothers as they bond with their infants.

## 2. Method

### 2.1. Study Design

We used a cross‐sectional, sequential explanatory mixed‐methods design, first collecting quantitative data through structured surveys and then conducting qualitative interviews with mothers with and without PPDS. This design allowed qualitative findings to clarify and enhance the quantitative results, deepening our understanding of factors influencing mother–infant bonding in PPDS cases [18]. Ethical approval was granted by the Ghana Health Service Ethics Review Committee (GHS‐ERC: 016/06/24).

Study methods, including the study sites, recruitment process and data‐collection procedures, followed the approach previously described in Tornyevah et al. [19]. Briefly, the study took place from September to November 2024 at Ho Polyclinic, Ho Municipal Hospital and Ho Teaching Hospital, representing primary, secondary and tertiary maternal care in the Volta Region of Ghana. The study included mothers aged 18 and older whose most recent live birth occurred within the past 12 months of the survey. Postnatal and immunisation clinic attendees (September–November 2024) were invited. Trained research assistants explained the study, provided information sheets and invited eligible mothers. Those who declined or were ineligible were excluded. Of the 405 mothers approached, five were ineligible (one under 18, four with older infants), resulting in a 98.8% eligibility rate. All 400 eligible mothers participated (100% response). One record was later excluded due to missing data, leaving 399 mothers (99.8%) for analysis.

For the qualitative phase, we purposefully sampled 31 mothers: 19 with PPDS and 12 without. This sample was sufficient, as thematic saturation typically occurs after 12–15 interviews [20].

### 2.2. Data Collection

After receiving written informed consent, research assistants conducted face‐to‐face interviews using structured questionnaires in Ewe or English on an Android tablet. The survey assessed maternal sociodemographic characteristics, emotional well‐being and the quality of mother–infant bonding. Data were entered into Castor EDC (v2024.3.4.0; https://www.castoredc.com/), hosted by Amsterdam University Medical Centre. Built‐in checks ensured data quality. Electronic entry enhanced confidentiality and reduced social‐desirability bias [21, 22]. The questionnaire was pilot‐tested with 10 mothers, and minor wording adjustments were made for clarity.

The survey was followed by interviews to provide additional context. Mothers were grouped based on their PHQ‐9 scores: above 9 (PPDS) or below 9 (without PPDS). Trained assistants contacted mothers, explained the study and scheduled interviews. These interviews were conducted privately in a separate room within the health facilities, with only the interviewer and the mother present. Infants attended if the mother was breastfeeding. Before participating, mothers provided written consent and were assured that their involvement would not affect their care. Mothers with positive PPDS screening results were encouraged to see a clinician or nurse before their interview. A semistructured interview guide was developed using insights from previous studies and based on the research objectives. It explored mothers’ views on bonding with their infants, caregiving, available support and the influence of cultural or religious beliefs.

Two postpartum mothers from a non–study site participated in piloting the questions to assess their clarity, flow and order before data collection. Each interview lasted 30–45 min and was recorded with permission on a Sony ICD digital voice recorder. Field notes documented contextual observations and early analyses. The research team reviewed interviews repeatedly and determined thematic saturation when no new ideas emerged. Research assistants summarised and verified participants’ views during interviews instead of returning transcripts to avoid adding extra burdens on mothers. Their knowledge of local languages and customs helped build trust and encouraged open discussion [23]. Each mother received GHS 45 (≈USD 3) as a token of appreciation for her time in the surveys and interviews.

#### 2.2.1. PPDS

The exposure of interest was PPDS, measured using the Patient Health Questionnaire‐9 (PHQ‐9), which has been validated in Ghana (Kintampo District; Cronbach’s *α* = 0.75) [24]. The PHQ‐9 demonstrates good screening performance, with a sensitivity of 0.84 and specificity of 0.81 for detecting depressive symptoms [25]. We used the PHQ‐9 because it has been validated in Ghana and is widely used in both general and maternal mental health research in similar settings [24, 25]. In addition, evidence from meta‐analysis indicates that the PHQ‐9 performs well as a screening tool for perinatal depression and has diagnostic accuracy comparable to that of the Edinburgh Postnatal Depression Scale (EPDS), which was specifically developed for postpartum populations [26, 27]. However, we acknowledge that the EPDS is a postpartum‐specific instrument and that its use may have offered additional contextual specificity. The PHQ‐9 comprises nine items with four response options (0 = Not at all; 1 = Several days; 2 = More than half the days; 3 = Nearly every day), yielding a total score ranging from 0 to 27 (e.g., “over the last 2 weeks, how often have you had little interest or pleasure in doing things?”). In this study, being at risk of postpartum depression was defined as a PHQ‐9 score above 9, indicating moderate or higher levels of depressive symptoms [25, 27].

#### 2.2.2. Maternal Bonding

The main outcome was the mean mother–infant bonding score, assessed using the 5‐item, 4‐point Pre‐ and Postnatal Bonding Scale (PPBS) (0 = Not at all, 1 = Hardly, 2 = Much, 3 = Very much; e.g., “Over the past 4 weeks, I can describe my feelings toward my baby”). Higher scores indicate better bonding [28]. The internal consistency of the PPBS in our study was acceptable (Cronbach’s *α* = 0.80).

#### 2.2.3. Covariates

A directed acyclic graph (DAG) was developed to clarify the causal assumptions underlying the relationship between the exposure and the outcome. First, the primary exposure and outcome of interest were identified. Second, risk factors for the outcome were gathered through a review of relevant literature. From this pool, variables that also served as risk factors for the exposure were identified as potential confounders. Third, the relationships among these variables and their common ancestors (variables that affect both exposure and outcome) were mapped based on theoretical and empirical evidence. Finally, the completed DAG was examined to identify all backdoor (biasing) paths between the exposure and outcome, and these paths were blocked by adjusting for the appropriate variables, ensuring that no cyclic paths were created. The DAG was created using DAGitty software (version 3.1; https://www.dagitty.net).

**FIGURE 1:**
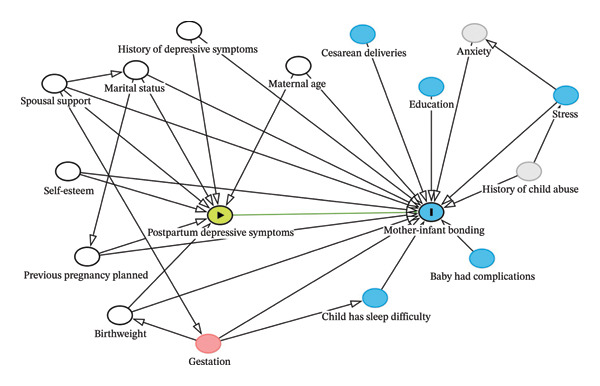
Directed acyclic graph (DAG) for the association between postpartum depressive symptoms and mother–infant bonding. Green circle with triangle = exposure of interest (postpartum depressive symptoms); blue circle with “I” = outcome of interest (mother–infant bonding); green line = causal path; white circles = adjusted variables; blue circles = ancestor of mother–infant bonding; grey circles = unobserved (latent) variables; red circle = ancestor of exposure and outcome.

Based on the DAG (Figure 1), we included relevant variables as potential confounders for adjustment in our model, including sociodemographic (maternal age, education), obstetric (parity, pregnancy intention), psychosocial (spousal support), psychological (self‐esteem, history of depressive, mood or other psychiatric disorders) and infant‐related factors (sex, birth weight, birth complications) [29, 30].

Self‐esteem was measured using the Rosenberg Self‐Esteem Scale (RSE). The RSE is a 10‐item questionnaire with four response options: strongly agree, agree, strongly disagree and disagree. The total score (ranging from 0 to 30) was calculated after reverse‐scoring of the five negatively worded items, with higher scores indicating greater self‐esteem. An RSE score below 15 indicates low self‐esteem. [31, 32]. The RSE has shown good internal consistency in prior studies (Cronbach’s *α* = 0.82) [33].

### 2.3. Data Analysis

For each group based on PPDS screening status (scores > 9), background characteristics were summarised using frequencies and proportions for categorical variables, while median and interquartile ranges were used for continuous variables. Pearson chi‐squared tests or Fisher’s exact tests, as appropriate, were used to assess differences in the distribution of background characteristics by PPDS screening status. The log‐normal multivariable regression model was used to estimate the association between risk of PPDS and mean bonding score, modelled on a continuous scale, adjusting for potential confounders identified based on a DAG (Figure 1). All statistical analyses were performed using Stata 19 MP4 (StataCorp, College Station, TX, USA).

Interview data were analysed thematically using Braun and Clarke’s reflexive approach [34] as previously described by Tornyevah et al. [19]. Leticia Tornyevah first immersed herself in the transcripts and field notes to gain a holistic understanding of participants’ experiences. A coding framework was then developed, combining inductive insights from the data with deductive attention to concepts identified from the literature and quantitative findings. Using Dedoose version 9.0 (Socio‐Cultural Research Consultants, LLC, Los Angeles, CA, USA), Leticia Tornyevah applied this framework systematically across all transcripts. Related codes were clustered into potential themes, which were refined through team discussions involving Anjali Sharma, Ank De Jonge, Jens Henrichs and Samuel Bosomprah until interpretive agreement was achieved. To ensure rigour, Anjali Sharma independently reviewed a subset of transcripts. The final themes were synthesised into a narrative that illustrates key maternal experiences with representative quotations.

The coding framework, with accompanying definitions and example quotations, was used to support transparency in analysis. Credibility was enhanced by linking qualitative themes with significant quantitative findings through a joint display and narrative summary [35]. Formal participant verification of findings was not undertaken to minimise participant burden; however, accuracy was ensured through in‐interview summaries and clarification probes. Including diverse and negative cases further strengthened dependability and confirmability. The quantitative results were reported according to STROBE guidelines [36], and the qualitative findings followed the COREQ checklist [37].

### 2.4. Research Team and Reflexivity

Reflexive principles guided the study. Interviewers kept field notes of assumptions and reflections. Regular peer review of these notes ensured transparency. Pre‐fieldwork training covered trauma‐informed, ethical interviewing and reflexive awareness. Role‐play enhanced interviewers’ communication, reflective skills and team consistency. Female fieldworkers fluent in Ewe and English conducted all interviews. None knew the participants. At recruitment, interviewers, including Leticia Tornyevah, introduced the study, explained its purpose and emphasised that participation was voluntary and there were no right or wrong answers. The team included Leticia Tornyevah (PhD candidate, maternal mental health), Samuel Bosomprah (PhD, biostatistics), Anjali Sharma (ScD, social science), Ank De Jonge (PhD, midwifery) and Jens Henrichs (PhD, perinatal mental health), from Ghana, the Netherlands and Zambia. Their expertise provided diverse perspectives, and frequent reviews of the coding and interpretation of qualitative data helped reduce bias [19].

## 3. Results

### 3.1. Background Characteristics of Participants

Survey data were analysed for 399 participating mothers. Most participants were aged 25–34 years (258, 64.7%), with a median age of 30 years (IQR = 26.0–34.0). Nearly two‐thirds (260, 65.2%) had completed at least secondary education, and four in five (322, 80.7%) worked in formal or self‐employment. The majority lived in urban areas (380, 95.2%), were married or cohabiting (343, 86.0%) and identified as Christian (377, 94.5%) (Table 1). Most had supportive partners (353, 88.5%) and no history of depressive or mood disorders (279, 69.9%) (Table 1). Most infants were born at term (363, 91.0%) with normal birth weight (356, 89.2%) (Table 2).

**TABLE 1:** Distribution of sociodemographic and social factors by PPDS status among mothers who gave birth within the last 12 months.

Characteristics	Screen negative for PPD, *n* (% of total)	Screen positive for PPD, *n* (% of total)	*p* value
*N*	282 (70.7%)	117 (29.3%)	
Age (years)	31 [26; 34]	29 [25; 33]	0.035
Age group (years)			
18–24	40 (14.2)	28 (23.9)	0.056
25–34	187 (66.3)	71 (60.7)	
≥ 35	55 (19.5)	18 (15.4)	
Education			
None/primary	88 (31.2)	51 (43.6)	0.018
Secondary+	194 (68.8)	66 (56.4)	
Occupation			
Employed (formal/self)	234 (83.0)	88 (75.2)	0.074
Unemployed	48 (17.0)	29 (24.8)	
Location			
Urban	273 (96.8)	107 (91.5)	0.022
Rural	9 (3.2)	10 (8.5)	
Marital status			
In union	253 (89.7)	90 (76.9)	< 0.001
Not in union	29 (10.3)	27 (23.1)	
Religion			
Christian	263 (93.3)	114 (97.4)	0.096
Other	19 (6.7)	3 (2.6)	
Spousal support			
Supportive	268 (95.0)	85 (72.6)	< 0.001
Unsupportive	14 (5.0)	32 (27.4)	
History of depressive/mood/psychiatric disorder			
No	228 (80.9)	51 (43.6)	< 0.001
Yes	54 (19.1)	66 (56.4)	

**TABLE 2:** Distribution of maternal and paediatric factors by PPDS status among mothers who gave birth within the last 12 months.

Characteristics	Screen negative for PPD, *N* (% of total)	Screen positive for PPD, *N* (% of total)	*p* value
Family history of psychiatric illness			
No	272 (96.5)	109 (93.2)	0.149
Yes	10 (3.5)	8 (6.8)	
Previous pregnancy planned			
Yes	186 (66.0)	41 (35.0)	< 0.001
No	96 (34.0)	76 (65.0)	
Type of pregnancy			
Singlet pregnancy	273 (96.8)	108 (92.3)	0.049
Multiple pregnancy	9 (3.2)	9 (7.7)	
Body mass index (BMI)			
Obese	84 (29.8)	32 (27.4)	0.040
Overweight	78 (27.7)	48 (41.0)	
Healthy weight	111 (39.4)	36 (30.8)	
Underweight	9 (3.2)	1 (0.9)	
Self‐esteem (RSE)			
Normal/high self‐esteem	279 (98.9)	93 (79.5)	< 0.001
Low self‐esteem	3 (1.1)	24 (20.5)	
Obstetric complications			
No	174 (61.7)	56 (47.9)	0.011
Yes	108 (38.3)	61 (52.1)	
Baby had complications at birth			
No	237 (84.0)	87 (74.4)	0.024
Yes	45 (16.0)	30 (25.6)	
Gestation			
Term	260 (92.2)	103 (88.0)	0.186
Preterm	22 (7.8)	14 (12.0)	
Birth weight			
Normal	259 (91.8)	97 (82.9)	0.009
Low	23 (8.2)	20 (17.1)	

Mothers who screened positive for PPDS were younger (median = 29 vs 31 years), less educated (56.4% vs 68.8%) and more likely to live in rural areas (8.5% vs 3.2%) or to not be in a union (23.1% vs 10.3%) (Table 1). They were also more likely to have unsupportive partners (27.4% vs 5.0%) and a history of depressive or mood disorders (56.4% vs 19.1%) (Table 1). In addition, higher proportions of women with PPDS reported unplanned pregnancies (65.0% vs 34.0%), multiple pregnancy (7.7% vs 3.2%), overweight BMI (41.0% vs 27.7%) and obstetric complications (52.1% vs 38.3%), as well as infants with birth complications (25.6% vs 16.0%) or low birth weight (17.1% vs 8.2%) (all *p* < 0.05) (Table 2).

### 3.2. PPDS and Mother–Infant Bonding Score

Mothers who screened negative for PPDS had significantly higher mean mother–infant bonding scores (M = 14.78, SD = 0.74) than mothers at risk for postpartum depression (M = 13.95, SD = 1.72; unadjusted mean ratio = 0.94; 95% CI [0.93, 0.96], *p* < 0.001) (Cronbach’s *α* = 0.80) (Table 3). After adjusting for the potential confounding effect of maternal age, marital status, spousal support, history of depressive/mood/psychiatric previous pregnancy planned, self‐esteem, birthweight and baby had complications at birth, there was evidence of a reduction of 3% in mean maternal bonding score due to screening positive for being at risk of PPD (adjusted mean ratio [aMR] = 0.97; 95% CI [0.95–0.99]; *p* = 0.01) (Table 3).

**TABLE 3:** Association of maternal bonding score with PPDS among mothers who gave birth within the last 12 months.

Postpartum depressive symptoms	# Participants (% of total); *N* = 399	Mean (SD) bonding score	Unadjusted		Adjusted^∗^	
			Mean ratio [95% CI]	*p* value	Mean ratio [95% CI]	*p* value
Screen negative for PPDS	282 (71)	14.78 (0.74)	Ref		Ref	
Screen positive for PPDS	117 (29)	13.95 (1.72)	0.94 (0.93, 0.96)	< 0.001	0.97 (0.95, 0.99)	0.01

^∗^Estimates were adjusted for maternal age (continuous), marital status (binary), spousal support (binary), history of depressive/mood/psychiatric disorder (binary), previous pregnancy planned (binary), self‐esteem (binary), birthweight and baby had complications at birth (binary). Cronbach’s *α* = 0.80).

### 3.3. Qualitative Findings

Thirty‐one qualitative interviews were conducted; of these, 19 women had PPDS and 12 did not. The interviewed mothers broadly represented the overall survey sample. A thematic analysis identified four key themes related to bonding mentioned by mothers with and without PPDS. These themes included: (1) Maternal instinct and a sense of responsibility as protective factors, (2) Caregiving activities that strengthen bonds, (3) The role of social support systems in strengthening bonding and (4) Religious, cultural and societal expectations reinforcing maternal bonding. Although the emotional tone varied between groups, the underlying processes supporting bonding appeared similar, highlighting the resilience and universality of maternal connection across different emotional states in this context.

#### 3.3.1. Maternal Instinct and Sense of Responsibility as a Protective Factor

Mothers, regardless of their PPD status, expressed a profound sense of responsibility for their babies, consistently prioritising their infants’ well‐being above their own. This sense of duty served as a key protective factor, fostering resilience and strengthening the maternal bond. By focusing on their babies’ needs rather than their personal struggles, mothers often found emotional stability and fulfilment through caregiving. Many described happiness and gratitude for the deep connection they felt with their babies. Mothers experiencing PPD acknowledged emotional challenges that sometimes made bonding difficult. However, their maternal instinct and sense of duty continued to drive them to nurture and protect their infants. One mother shared, “*I may feel sad sometimes, but I always hold my baby and make sure she is okay. I love her no matter how I feel*” (P01, PPDS). Similarly reported by another mother, “*Even on days when I don’t feel like doing anything, I have to take care of my baby. She depends on me, and that keeps me goin*g” (P09, PPDS).

By contrast, mothers without PPDS described a more effortless and joyful bond with their babies, marked by affection, gratitude and emotional fulfilment. As one mother expressed, “*When I hold my baby and see him smile, it gives me so much joy. I can’t describe it. Bonding with him comes naturally; I just love being with him*” (P03, No PPDS). Another mother mentioned, “*Day by day I feel more connected to her; it feels natural and strong*” (P06, No PPDS). A third mother described increased bonding and how her baby keeps her company: “*Day in, day out, the bond grows. I feel happy because I was once lonely, and now I have them*” (*P05,* No PPDS).

These narratives illustrate how maternal instinct and a deep sense of responsibility serve as vital protective factors, fostering emotional resilience and strengthening maternal bonds. While mothers with PPDS often need greater intentional effort to connect, their instinctive commitment to caregiving remains central to sustaining the mother–infant relationship.

#### 3.3.2. Caregiving Activities Strengthen Maternal Bonding

Caregiving activities such as breastfeeding, skin‐to‐skin contact, bathing, playing and praying emerged as powerful facilitators of bonding across both groups. Mothers described these daily routines as emotionally fulfilling, reinforcing their connection with their infants.

For mothers with PPDS, caregiving provided emotional reassurance and served as a coping mechanism. These activities reminded them of their babies’ dependence on them, creating moments of relief and joy despite emotional distress. One mother shared, “*She depends on me for everything, so even if I don’t feel great, I hold her and feed her, and she smiles at me, and that keeps me going*” (P05, PPDS). Furthermore, another mother shared how carrying her baby provided emotional relief amidst her personal struggles. “*When I feed him, I feel good; it makes me forget my problems for a whi*le.” (P13, PPDS).

Mothers without PPDS echoed these sentiments, describing caregiving as joyful and rewarding. One mother said, “*By feeding him, carrying him around, and sometimes lying on my lap, I feel good*” (P12, No PPDS). Another mother shared, “*I love bathing him and playing with him. Those moments make me feel so happy*” (P07, No PPDS). Another mother highlighted the significance of prayer as a bonding activity*.* “*When I pray with my baby, I feel like we are connecting on a deeper level*” (P10, No PPDS).

These accounts demonstrate that caregiving activities not only nurture the baby but also provide mothers with emotional fulfilment and reassurance. For mothers with PPDS, caregiving may require more deliberate effort, but it remains a vital source of connection and emotional healing during the postpartum period.

#### 3.3.3. The Role of Social Support System in Strengthening Maternal Bonding

Mothers consistently highlighted the importance of social support from family, spouses and healthcare workers in sustaining a strong mother–infant bond. Emotional and practical support reduced postpartum stress and fostered resilience, particularly for mothers with PPDS. One mother explained, “*I know I have postpartum depression, but my mother-in-law helps me a lot. She encourages me, so I don’t feel like I’m failing as a mother*” (P14, PPDS). Describing the role of partner support, which facilitated bonding, a mother shared, “*My husband makes sure I rest while he takes care of the baby. That really helps me feel more connected to my child*” (P12, PPDS). Similarly, the responsibilities of health workers in supporting maternal bonds are shared: “*The midwives taught me why I needed to take care of myself after the C-section. It helped me feel less confused about my emotions*” (P03, PPDS).

Even mothers without PPDS recognised the value of support in easing the challenges of early motherhood. One noted, “*Even though I don’t have depression, it’s still overwhelming sometimes. Having my mother around to help has made things much easier*” (P09, No PPDS). Another echoed, “*My husband has been there from day one … He even helps at night so I can sleep*” (P08, No PPDS). The importance of professional support was also emphasised “*The nurses advised on feeding and keeping the baby healthy, which helped strengthen the bond*” (P02, No PPDS).

Collectively, these experiences underscore that supportive relationships act as buffers against stress, promoting maternal confidence and emotional stability. For mothers with PPDS, such support is especially protective, helping them maintain nurturing relationships with their infants.

#### 3.3.4. Religious, Cultural and Societal Expectations Reinforcing Maternal Bonding

Mothers described how religious beliefs, cultural traditions and societal expectations reinforced their sense of duty and devotion to their babies. These frameworks fostered perseverance in caregiving, even during periods of emotional distress. For many, motherhood was viewed as both a divine gift and a moral obligation that demanded constant care and presence.

Mothers with PPDS emphasised how faith and cultural expectations strengthened their motivation to care for their infants. As one expressed, “*God gave me this child for a reason. Even if I struggle, I must take care of my blessing*” (P11, PPDS). One mother also described the role of spirituality in strengthening bonding. “*When I feel stressed, I pray with her, and it helps me feel calmer and more capable, making us connect better*” (P15, PPDS). Another mother noted, “*In my culture, a mother must always be there for her baby. No matter how I feel, I cannot fail in my role*” (P10, PPDS).

Mothers without PPDS shared similar sentiments, linking motherhood to gratitude and pride. One reflected, “*Becoming a mother is a blessing from God, and I feel honoured to fulfil this role*” (P04, No PPDS). Also mentioned by a different mother, “*My mother always told me that being a good mother is the most important thing a woman can do. I take that seriously*” (P06, No PPDS).

Across both groups, mothers viewed their roles through the lens of faith and cultural responsibility, which reinforced caregiving behaviours and emotional connection. These beliefs cultivated perseverance and gratitude, strengthening maternal bonding even amid emotional challenges.

## 4. Discussion

In this study, 29% of mothers screened positive for PPDS, and screening positive was associated with slightly lower mother–infant bonding scores. Although the association was statistically significant, the magnitude of the difference was modest, and its clinical importance should therefore be interpreted with caution. Bonding scores remained generally high in both groups. The qualitative findings provide useful context for this pattern, suggesting that mothers maintained connection with their infants through maternal instinct and responsibility, caregiving and support from family, partners and health workers, as well as religious and sociocultural expectations of motherhood, even in the presence of emotional distress. Together, these findings suggest that while PPDS may affect emotional experiences, it does not necessarily disrupt mother–infant bonding. Instead, emotional resilience, caregiving and sociocultural influences may help buffer its impact.

Maternal bonding scores were slightly lower among mothers with PPDS. However, this difference remained modest, as mothers actively maintained the maternal bond despite emotional distress, drawing on instinct and a strong sense of moral responsibility. In this study, depressive symptoms were associated with reduced emotional closeness without disrupting the bond. Evidence from a systematic review similarly shows that depressive symptoms are associated with slightly lower but generally preserved bonding [38], with comparable patterns reported by Cuijlits [28] and Reck et al. [39]. In contrast to studies that conceptualise bonding as primarily emotion‐driven [2, 40], mothers in this study deliberately nurtured their infants despite these challenges, with a moral sense of purpose driving continued engagement. Rather than a passive reflection of maternal affect, it is tempting to speculate that bonding may be viewed as an actively sustained process shaped by emotional labour, moral responsibility and relational commitment [41], offering a perspective that may complement emotion‐centred and deficit‐based models. Ghanaian studies similarly highlight how moral obligation and gratitude help maintain the mother–infant bond despite psychological distress [42, 43]. Collectively, the integrated findings suggest that maternal instinct and moral responsibility may function as important emotional resources that help anchor the mother–infant bond, helping preserve it even when emotional connection is strained.

Bonding remained relatively high among mothers with PPDS, with caregiving playing a central role in sustaining this bond despite emotional strain. Mothers described these activities as both a coping strategy and an expression of affection that restored calm and closeness with their infants. Daily activities such as feeding, bathing and playing transformed moments of sadness into opportunities for connection, reinforcing the bond, while infant responses such as smiles or calmness further restored mothers’ sense of competence and joy. This aligns with evidence that sensitive caregiving supports mother–infant synchrony, although depressive symptoms may reduce emotional cues [44, 45]. Maternal responsiveness can persist despite depressive symptoms, suggesting that such engagement serves as an adaptive pathway for maintaining bonding, with studies in Ghana and similar settings showing maternal care practices as a coping strategy for psychological distress [46, 47]. Overall, caregiving may be understood as both practical support and an emotional bridge, helping mothers re‐establish confidence and maternal identity amid psychological strain.

Beyond individual resilience, broader social, religious and cultural contexts also reinforced bonding and provide important context for the modest differences observed. Despite slightly lower bonding scores among mothers with PPDS, mothers emphasised that they received encouragement and practical assistance from partners, relatives and health workers, which helped them manage fatigue and anxiety while maintaining infant care and emotional connection. This resonates with evidence that social support strengthens bonding and mitigates the impact of PPDS on mother–infant relationships [41, 48]. Empathic guidance from midwives and nurses helped mothers navigate emotional changes and provided reassurance after childbirth, underscoring the importance of sensitive postpartum care [49]. Faith and cultural traditions also provided meaning and hope that supported continued nurturing, with motherhood viewed as both a divine calling and a valued social role. These expectations encouraged attentiveness and emotional engagement even under distress, consistent with studies from Ghana and other sub‐Saharan contexts showing that spirituality and cultural norms reinforce maternal commitment and responsiveness, although such ideals may also create guilt when positive emotions are lacking [42, 43, 47]. Taken together, the mixed‐methods findings suggest that while PPDS was associated with slightly lower bonding scores, this association was tempered by contextual and relational factors identified in the qualitative data. Mothers described caregiving routines, social support and cultural and religious expectations as important resources that helped maintain connection with their infants despite emotional distress. The qualitative insights therefore provide an important interpretive context for the modest quantitative effect observed.

This study has several strengths and limitations. The mixed‐methods design allowed us to quantify the association between PPDS and mother–infant bonding while also exploring how mothers understood and sustained bonding in their daily lives. However, several limitations should be considered. First, the cross‐sectional design precludes causal inference. Second, PPDS and bonding were assessed using self‐report measures, which may be influenced by social desirability and recall bias. We also did not use a postpartum‐specific screening instrument such as the EPDS; although the PHQ‐9 has demonstrated adequate screening performance for perinatal depression, the use of a postpartum‐specific instrument may have provided greater contextual sensitivity. Third, the PPBS has not yet been formally validated in Ghana, and culturally specific expressions of bonding may not be fully captured by this measure, as bonding in this context may be expressed not only through emotional closeness but also through relational commitment, caregiving practices and socially embedded expectations of motherhood. Fourth, bonding scores were high across participants, raising the possibility of a ceiling effect in the PPBS. Restricted variability in the measure may have attenuated observable differences between mothers with and without PPDS and should be considered when interpreting the modest effect size. Finally, interviewers were not blinded to participants’ PPDS status during the qualitative phase, which may have introduced interviewer bias. However, the use of a semistructured interview guide and interviewer training helped to promote consistency across interviews and reduce subjective influence.

## 5. Conclusion and Relevance for Clinical Practice

We found a small but statistically significant association between PPDS and lower mother–infant bonding scores, although bonding remained generally high in both groups. Qualitative findings suggested that mothers drew on maternal instinct and a sense of responsibility, caregiving practices and social, cultural and spiritual support to sustain connection with their infants. These findings indicate that support for mothers with depressive symptoms should address emotional distress while also strengthening the contextual resources that may help preserve mother–infant bonding.

Findings from this study suggest that PPDS is associated with a modest reduction in mother–infant bonding, with overall bonding remaining high. Mothers appeared to demonstrate resilience through caregiving, faith, social support and cultural expectations that supported emotional connection despite stress. For nurses and midwives, these findings highlight the importance of recognising and strengthening mothers’ existing coping strategies and support networks. Providing culturally and spiritually sensitive, strength‐based care may help support emotional well‐being and healthy mother–infant relationships.

## Author Contributions

Leticia Tornyevah, Samuel Bosomprah, Jens Henrichs and Ank De Jonge conceptualised the study. Leticia Tornyevah, Anjali Sharma and Samuel Bosomprah analysed the data, and all authors interpreted it. Leticia Tornyevah drafted the manuscript. All authors reviewed the draft.

## Funding

This research was funded in whole or in part by Science for Africa Foundation to the Developing Excellence in Leadership, Training and Science in Africa (DELTAS Africa) programme (DEL‐22‐012) with support from Wellcome Trust and the UK Foreign, Commonwealth & Development Office and is part of the EDCPT2 programme supported by the European Union. For purposes of open access, the author has applied a CC BY public copyright licence to any Author Accepted Manuscript version arising from this submission.

## Disclosure

All authors read and approved the final version.

## Conflicts of Interest

The authors declare no conflicts of interest.

## Data Availability

Data will be provided to any interested researchers upon request. To access the data, contact the corresponding author and specify the intended use. A data request should include contact details, a research project name, a description of the proposed analysis and the expected format. The data should only be used for purposes related to the original research or study.
